# Feeding Agro-Industrial By-Products to Light Lambs: Influence on Meat Characteristics, Lipid Oxidation, and Fatty Acid Profile

**DOI:** 10.3390/ani10091572

**Published:** 2020-09-03

**Authors:** Trinidad de Evan, Almudena Cabezas, Jesús de la Fuente Vázquez, María Dolores Carro

**Affiliations:** 1 Departamento de Producción Agraria, ETSIAAB, Universidad Politécnica de Madrid, Ciudad Universitaria, 28040 Madrid, Spain; t.deevan@alumnos.upm.es; 2 Departamento de Producción Animal, Facultad de Veterinaria, Universidad Complutense de Madrid, 20840 Madrid, Spain; almucabe@ucm.es (A.C.); jefuente@vet.ucm.es (J.d.l.F.V.)

**Keywords:** meat quality, fatty acid, meat oxidation, corn distillers dried grains with solubles, citrus pulp, exhausted olive cake

## Abstract

**Simple Summary:**

Consumers demand safe and healthy animal products produced with minimal environmental impact. The use of agro-industrial by-products in animal feeding can alleviate pollution caused by their accumulation, but investigations into their effects on animal-product quality are required. We analyzed the influence of replacing 44% of conventional feeds in a high-cereal concentrate (CON) with agro-industrial by-products (BYP concentrate; distillers dried grains with solubles, dried citrus pulp, and exhausted olive cake) on meat quality of fattening light lambs. Two groups of lambs were fed each of the concentrates and barley straw ad libitum from 13.8 to about 26.0 kg of body weight. The pH, chemical composition, color, and texture parameters of the meat were not affected by the type of concentrate. Feeding the BYP concentrate significantly reduced lipid oxidation of meat after 6 days of refrigerated storage, which might be related to the greater content of polyphenols in this concentrate. Compared with CON-fed lambs, the meat from BYP-fed lambs had lower saturated and greater polyunsaturated fatty acid content. In summary, feeding the tested by-products did not change the composition of the meat but increased its shelf-life and improved its fatty acid profile.

**Abstract:**

The aim of this study was to assess the effects of replacing 44% of conventional feeds in a high-cereal concentrate (CON) with by-products (BYP concentrate; 18% corn distillers dried grains with solubles, 18% dried citrus pulp, and 8% exhausted olive cake) on the meat characteristics and fatty acid (FA) profile of fattening light lambs. Two groups of 12 Lacaune lambs were fed concentrate and barley straw ad libitum from 13.8 to 26.0 kg of body weight. There were no differences (*p* ≥ 0.130) between groups in the pH, chemical composition, color, and texture parameters and in the estimated proportions of pigments in the longissimus dorsi. Feeding the BYP concentrate reduced the concentration of thiobarbituric acid reactive substances (TBARS) in the meat after 6 days of refrigerated storage (unmodified atmosphere), probably due to the greater polyphenol content in this concentrate. Compared with CON-fed lambs, the meat and the subcutaneous fat from BYP-fed lambs had lower saturated and greater polyunsaturated FA content as well as greater n-6/n-3 FA. In summary, feeding a blend of corn distiller dried grains with solubles, dried citrus pulp, and exhausted olive cake did not change the composition of the meat but improved its antioxidant status and FA profile.

## 1. Introduction

Compared with other livestock productions such as poultry and pork meat, ruminant meat is associated with both a greater environmental cost and consumer perception of a less healthy product, mainly due to its high content in saturated fat [1]. The use of agro-industrial by-products in ruminant diets not only contributes to reducing environmental problems caused by the accumulation of by-products and to decreasing animal product carbon footprints [2] but also can improve the quality of products and the meat shelf-life stability [1,3] due to the presence of bioactive compounds in some by-products, like vitamins, unsaturated fatty acids (FA), and phytochemicals.

Recently, de Evan et al. [4] reported that 44% of conventional feeds in a high-cereal concentrate for fattening lambs can be replaced by a mixture of corn dried distiller grains with solubles (DDGS), dried citrus pulp (DCP), and exhausted olive cake (EOC) without affecting lamb performance. Both DCP and EOC are rich in polyphenols and other bioactive compounds [5,6,7], and therefore, they might improve lamb meat composition and might increase meat shelf-life stability [8,9]. In addition, DDGS are by-products of the ethanol industry rich in unsaturated FA and their inclusion in the diet can modify the lipid composition, oxidative stability, and sensory characteristics of lamb meat [10]. The polyphenols in the by-products can also modify the rumen biohydrogenation of the unsaturated FA, resulting in improved quality of the fat of animal products. Some studies have investigated the effects of each of these by-products on lamb performance, but information on their effects on meat quality is more limited. Our hypothesis was that feeding a combination of DDGS, DCP, and EOC to fattening light lambs may modify meat FA profiles towards a more unsaturated profile and may increase meat shelf-life stability. Therefore, the objective of this study was to evaluate the characteristics of the meat of lambs fed a concentrate containing these by-products.

## 2. Materials and Methods

This is a companion paper to the study of de Evan et al. [4], who reported the effects of by-product (BYP) concentrate on growth performance, diet digestibility, blood parameters, and ruminal fermentation of the lambs. The animals used in this experiment were cared for and handled in accordance with the Spanish guidelines for experimental animal protection. All experimental procedures, including slaughtering, were approved by the General Direction of Livestock and Agriculture of the Community of Madrid (approval number PROEX 035/17).

### 2.1. Diets and Animals

Diets and animals are described in detail by de Evan et al. [4] and are only briefly summarized here. Two groups of 12 Lacaune male lambs each (13.8 ± 0.25 kg initial body weight) were penned individually in 1 m × 1 m pens with slatted floor and equipped with two feeders and an automatic drinker. Each experimental group was randomly assigned to one of the two dietary treatments: a high-cereal concentrate (CON) and a concentrate including agro-industrial by-products (BYP). The ingredients, chemical composition, and FA profiles of both concentrates are shown in Table 1. Both concentrates had similar content in neutral detergent fiber (NDF), but the crude protein (CP) and ether extract (EE) contents were greater in the BYP than in the CON concentrate.

### 2.2. Slaughter Procedure and Sampling

The experiment lasted for six weeks, and lambs were slaughtered at about 26 kg of body weight in two different days (6 lambs of each treatment per day) at a commercial slaughterhouse located 20 km away from the experimental farm. The slaughter procedure followed commercial practices in Spain and involved head electrical stunning and severing of the carotid arteries and jugular veins. After slaughter and dressing, the pH of the longissimus dorsi (at the thoracic vertebrae (T) T13 rib level) and semitendinosus muscles was measured using a penetration electrode adapted to a portable pH meter with a temperature probe (Hanna Instruments pH meter HI-9025; Hanna Instruments SL, Eibar, Spain). The measurements were repeated after 24 h of chilling at 4 °C, and two measurements were made for each muscle at each time. In addition, color was measured on the subcutaneous fat of the tail root at 24 h after slaughter, and one sample of this fat was taken and frozen (−20 °C) until analysis of FA profiles.

The whole longissimus dorsi from the left side of the carcass of each lamb was dissected and divided in several pieces. The portion between T12 and T13 (about 2 cm) was immediately frozen (−20 °C) and stored before freeze-drying and chemical composition analyses. The piece between T13 and the lumbar vertebrae (L) L6 (about 10 cm length) was vacuum-packed and frozen (−20 °C) until texture analysis. The portion between T9 and T12 was used to assess the evolution of color, lipid oxidation, and FA profile over a 6-day ageing period and was subdivided into 3 equal pieces (about 25 g each). The color of the samples taken for analyses of initial time (day 0) was measured, and samples were then vacuum-packed and frozen (−20 °C) for analyses of lipid oxidation and FA profile. The 2 remaining portions from each lamb were placed on Styrofoam trays, which were overwrapped with an oxygen-permeable polyvinyl chloride film and stored in the dark at 2 °C until analyses. Color was measured after 1 h of blooming, and after that, samples were immediately vacuum-packed and frozen (−20 °C) for further analyses of lipid oxidation (3- and 6-day storage samples) and FA profile (6-day storage samples).

### 2.3. Sensory Characteristics and Meat Shelf Life

The color was evaluated using a CM-2500c Minolta Spectophotometer (Minolta Co., Osaka, Japan) with illuminant D65, visual angle 10°, and 8-mm measurement aperture. The calibration was performed as described by de la Fuente-Vázquez et al. [11] using standard white tiles prior to color measurements. The color was measured three times on each sample, and values were averaged before statistical analysis. The color coordinates were expressed by the CIELAB system [12] as L* (brightness), a* (red-green index), and b* (yellow-blue index). Chroma (C*) and hue angle (h*) values were calculated as C* = (a*^2^ + b*^2^)^0.5^ and h* = tan^−1^ (b*/a*), respectively. In addition, the myoglobin, metmyoglobin, and oxymyoglobin proportions on the meat surface were estimated by measuring the absorbances (A) at 473, 525, 572, and 690 nm according to the procedure of Krzywicki [13] with some modification proposed for lamb meat by de la Fuente-Vázquez et al. [11]. The values were calculated as myoglobin (%) = 2.375 × (1 − ((A_473_ − A_690_)/(A_525_ − A_690_))) × 100, metmyoglobin (%) = (1.395 − ((A_572_ − A_690_)/(A_525_ − A_690_))) × 100, and oxymyoglobin (%) = 100 − (myoglobin + metmyoglobin).

Texture analyses were performed on longissimus dorsi samples (about 30 g) as described by Díaz et al. [14]. Briefly, samples were thawed at 4 °C, removed from their packaging and blotted dry without pressing, and cooked into air bags in a water bath (75 °C, 30 min). After that, samples were cooled at room temperature for 30 min and removed from the cooking bags, and the exudate juice was gently cleaned before cutting the samples into pieces (1 × 1 cm cross section; 2 cm length) parallel to the muscle fiber direction. Texture was then measured using a TA-XT2 Texture Analyser ^®^ (Stable Micro Systems, Surrey, UK) equipped with a Warner–Bräzler blade. The parameters measured were the maximum shear force (newton/cm^2^), shear firmness (newton/s cm^2^), and total area defined as the total work performed to cut the sample or the area under the curve (toughness; newton s/cm^2^).

Lipid oxidation of longissimus dorsi samples at 0, 3, and 6 days of storage was assessed by measuring the concentrations of thiobarbituric acid reactive substances (TBARS) and conjugated dienes (CDs). The TBARS concentration was measured as described by Maraschiello et al. [15], and the results were expressed as g malonaldehyde (MAE)/kg meat. Conjugated dienes (CDs) were analyzed in the same samples according to the method described by Sirinivasan et al. [16] with the modifications proposed by Juncachote et al. [17], and their concentration was expressed as μmol/g of meat using the molar extinction coefficient of 25,200 M^−1^ cm^−1^. Both analyses were conducted in duplicate, and the absorbance of extracts was measured in a Thermo Scientific Evolution 220 spectrophotometer (Thermo Fisher Scientific, Madrid, Spain) at 532 and 233 nm for TBARS and CD, respectively.

### 2.4. Analyses of Chemical Composition and Fatty Acid Profile

For analysis of moisture content in the longissimus dorsi, 5 g of muscle was homogenized in a crucible with sea sand, 5 mL of ethanol was added, and the samples were dried at 102 °C. The ash and ether extract contents of freeze-dried samples of longissimus dorsi were analyzed in duplicate following the Association of Official Analytical Chemists [18] procedures (ID 048.13 and 945.16, respectively). The nitrogen (N) content was assessed by the Dumas combustion method using a Leco FP258 N Analyzer (Leco Corporation, St. Joseph, MI, USA). The content in total soluble polyphenols in both concentrates was analyzed using the spectrophotometric Folin Ciocalteu assay as described by Singleton and Rossi [19], and results were reported as gallic acid equivalents (GAE). Other chemical analyses of the concentrates have been described by de Evan et al. [4].

The lipids from lyophilized samples of longissimus dorsi (200 mg) at 0 and 6 days of storage were extracted in duplicate as described by Segura and López-Bote [20]. Briefly, samples were homogenized in dichloromethane-methanol (8:2; vol/vol) using a mixer mill (MM400; Retsch technology, Stuttgart, Germany) and centrifuged (8 min, 10,000 rpm). The solvent was evaporated under a nitrogen stream, and the lipids were dried by vacuum desiccation before weighting for total lipid content determination. Fatty acid methyl esters were prepared by transesterification using a mixture of sodium methylate–methanol and were methylated in the presence of sulfuric acid as detailed in Segura and López-Bote [20]. The fatty acid methyl esters were separated using a gas chromatograph (HP 6890 Series GC System; Hewlett Packard Co., Avondale, PA, USA) equipped with a flame ionization detector and an HP-Innowax polyethylene glycol column (30 m × 0.316 mm × 0.25 µm; J&W Scientific/Agilent Technologies, Santa Clara, CA, USA) and using nitrogen as a carrier gas. Results were expressed as percentage of total FA identified. The same method was used to analyze the FA profile of subcutaneous fat of the tail root. The extraction of lipids from feeds (concentrate samples; 200 mg) followed the procedure of Sukhija and Palmquist [21] as detailed by Rodríguez et al. [22], and the preparation and identification of fatty acid methyl esters were conducted as described before.

### 2.5. Statistical Analyses

Normal distribution of data was assessed by the Shapiro–Wilk test [23]. Data obtained at a simple time point (chemical composition of meat, color, fatty acid profile, and health indexes of the subcutaneous fat) were analyzed as a one-way ANOVA using PROC GLM of the Statistical Analysis System [24], in which the diet was the main effect and lamb was the experimental unit. Data measured over time (pH, color, lipid oxidation parameters, FA profile, and health indexes of meat) were analyzed with the PROC MIXED of Statistical Analysis System [24] as a mixed model with repeated measures, in which the diet, sampling time, and their interaction were considered fixed effects and either the lamb or its meat was a random effect. Significance was declared at *p* < 0.05, and trends were declared at *p* < 0.10. When a significant effect of time was detected, means were compared by the Tukey test.

## 3. Results and Discussion

The present study analyzes the quality of meat from lambs fed either a high-cereal concentrate or a concentrate containing corn DDGS, DCP, and EOC. Previously, de Evan et al. [4] showed that there were no differences between concentrates in growing performance, digestibility of nutrients, and animal health of the lambs. There were also no differences in hot and cold carcass weights, which reached 14.4 and 13.6 kg for the CON and 14.2 and 13.6 kg for the BYP groups, respectively [4]. The greater content of total soluble polyphenols in the BYP compared with the CON concentrate was attributed to the inclusion of DCP and EOC. The EOC contained 2.03% of total soluble polyphenols (dry matter (DM) basis), which is in agreement with the values ranging from 0.4 to 2.9% reported by others for EOC samples [7,25]. Likewise, total soluble polyphenol content of the DCP used in our study (1.33%; dry matter basis) was similar to the values reported by Gorinstein et al. [26] but slightly greater than the values reported by others [27,28]. It has been shown that the content in total soluble polyphenols of DCP is highly variable, as it varies with the type of citrus fruit (lemon, orange, etc.) and the fruit fraction, being greater in the peels than in the pulp of the fruit [26,29].

The main differences between concentrates in the FA profile were observed in the contents of lauric (C12:0) and myristic (C14:0) acids, which were lower in the BYP than in the CON concentrate, and in the oleic acid (C18:1 n-9), which was greater in the BYP concentrate. Compared with CON, the BYP concentrate contained less saturated FA (SFA; 36.0 vs. 25.4%, respectively). This is in agreement with previous studies in which diets including DDGS, DCP, or EOC were tested [8,9,10], and it was attributed to the high proportion of unsaturated FA in DDGS, DCP, and EOC [5,6,30].

One of the main factors determining meat quality is its pH, which influences the organoleptic characteristics of the meat [31]. As shown in Table 2, no differences between diets were observed in the pH of the longissimus dorsi and semitendinosus muscles, and no interactions of diet × time were detected. As expected, pH decreased (*p* < 0.001) at 24 h postmortem in both muscles, reaching values below 6.0 within the range of optimal commercial quality [32]. The pH values observed in this study are in good agreement with those previously reported for the meat of lambs fed high-concentrate diets and slaughtered at similar body weight [32,33,34] and for lambs receiving diets including the same by-products [35,36].

A bright red color in lamb meat is desirable and attractive for consumers [37]. Although meat color can be strongly affected by the diet, in our study, there were no differences (*p* ≥ 0.130) between diets in any color parameter or in the estimated concentrations of pigments (Table 3) and no diet × time interactions were detected (*p* ≥ 0.608), indicating that changes in color over the storage period were not affected by dietary treatment. These results are in accordance with the lack of differences between diets in pH and chemical composition of the meat. In contrast, some authors have reported a discoloration of beef meat when crude olive cake was included in the diet at greater levels than that used in the present study [38,39], and Lanza et al. [40] observed that the inclusion of 10% of orange pulp and 10% carob pulp in the diet of Barbaresca lambs decreased the lightness and intramuscular fat content of the meat.

Refrigerated storage of the meat affected all color parameters and the estimated concentrations of the pigments with the exception of h*, which remained unchanged (Table 3). Lightness (L*) increased from days 0 to 3, recovering the original values by day 6 of storage, whereas a*, b*, and C* increased by day 3, followed by a stabilization thereafter. Similar increases in b* and C* have been observed by Ripoll et al. [41] and de la Fuente-Vázquez et al. [11] during refrigerated storage in atmospheric conditions of lamb meat for 7 days. In contrast, Inserra et al. [42] reported significant decreases of a* and C* values in lamb meat after 6 days of refrigerated storage in atmospheric conditions. Several factors can influence the stability of the meat color under the same storage conditions, and the diet of the animals and the composition of the meat have been identified as the main ones [11,43], helping to explain differences among studies.

The color of the meat depends on many factors, but the concentration of myoglobin and its chemical state is one of the most important factors involved [43]. The oxymyoglobin is responsible for the bright-red color in meat, but its oxidation to brown-colored metmyoglobin during meat ageing leads to meat discoloration [11]. In agreement with previous studies [11,43], myoglobin decreased (*p* < 0.001) and both metmyoglobin and oxymyoglobin increased (*p* < 0.001) over the 6 days of storage for both dietary treatments. Every time meat is allowed to bloom, oxymyoglobin is formed [44], and that is in accordance with the observed increases in a * and b * coordinates at days 3 and 6 compared with day 0. Meat with more than 40% metmyoglobin has been reported to be downgraded by trained consumer panels [45,46], but in our study, the estimated proportions were lower than this level in all samples. All these results would indicate high color stability in the meat of lambs from both groups.

The diet had no influence (*p* ≥ 0.166) on chemical composition and texture parameters of the longissimus dorsi (Table 4). Chemical composition of the meat was within the range reported by others [11,34,47,48] for lambs from different breeds slaughtered at similar body weight. Our results agree with those from other studies reporting that inclusion of corn DDGS [10], DCP [49], or EOC [50] in the concentrate for fattening lambs had no effects on chemical composition of meat. Some studies reported that the inclusion of corn DDGS in the diet of Rambouillet lambs (20% DDGS [51]) and Wrzosówka lambs (45% DDGS [10]) resulted in a more delicate texture. Lanza et al. [40] observed that feeding 10% of orange pulp and 10% carob pulp to Barbaresca lambs increased the tenderness of their meat. Differences among studies in the composition and inclusion rate of the by-products, in the breed of the animals, and in their weight and age at slaughter can explain the discrepancies with our results.

**Figure 1:**
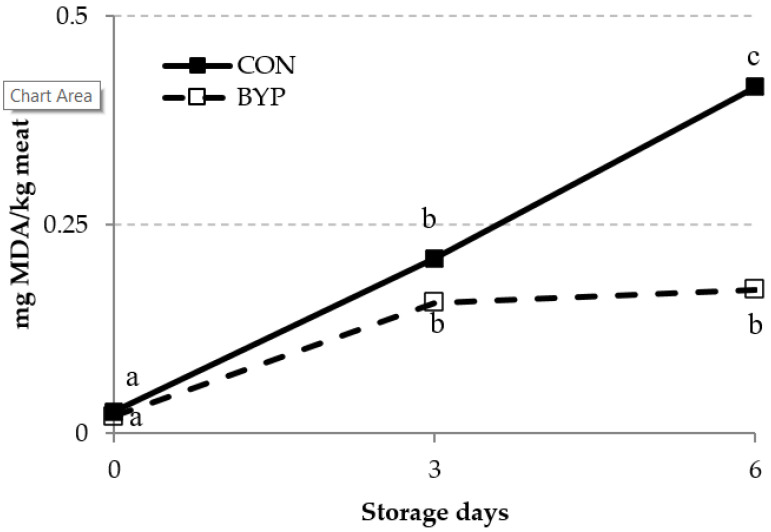
Effect of the dietary treatment (CON and BYP) and storage time (0, 3, and 6 days) on the concentration of TBARS (expressed as mg malonaldehyde (MDA)/kg meat) measured in lamb meat stored in unmodified atmosphere at 2 °C. ^a, b, c^ Within each dietary treatment, different superscripts indicate differences between days of storage (*p* < 0.05). In repeated measures ANOVA, values of *p* were 0.134, <0.001, and 0.095 for diet, time, and diet × time interaction, respectively, and standard error of the mean (SEM) values were 0.0326 and 0.0399 for diet and time effects, respectively.

**Figure 2:**
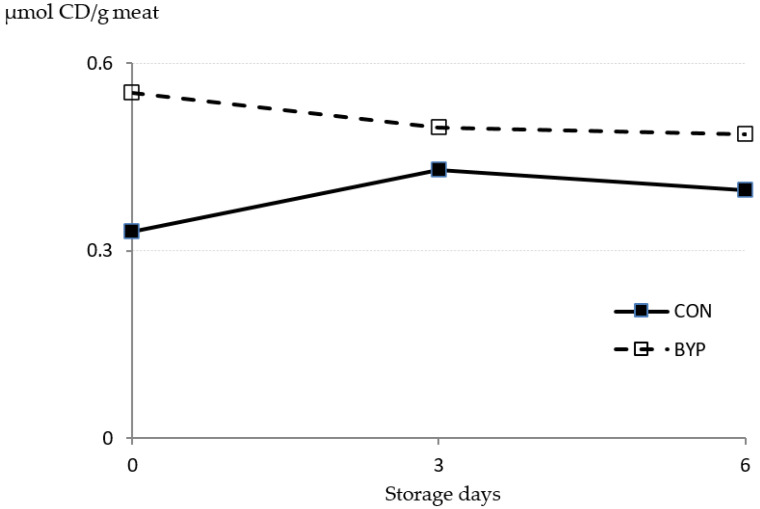
Effect of the dietary treatment (CON and BYP) and storage time (0, 3, and 6 days) on the concentration of conjugated dienes (CD; expressed as µmol/g meat) measured in lamb meat stored in unmodified atmosphere at 2 °C: in repeated measures ANOVA, values of *p* were 0.219, 0.642, and 0.740 for diet, time, and diet × time interaction, respectively, and standard error of the mean (SEM) values were 0.0265 and 0.0325 for diet and time effects, respectively.

The lipid oxidation of the meat over a 6-day storage period was assessed by measuring the concentrations of TBARS and CD (Figure 1 and Figure 2). The TBARS is one of the most widely used assays for measuring malondialdehyde, an end product of lipid peroxidation, whereas the CDs are primary lipid oxidation products generated in the oxidation of polyunsaturated FA (PUFA [52]). There was a trend (*p* = 0.095) for a significant diet × time interaction for TBARS concentration, but the diet did not affect TBARS levels in the meat (*p* = 0.134). Some authors [53,54,55] have reported that the dietary administration of antioxidants to fattening lambs reduced the accumulation of malondialdehyde concentration and improved the resistance of meat to oxidative deterioration. Feeding antioxidant-rich by-products to lambs is a feasible option to increasing the daily intake of antioxidants. In fact, Inserra et al. [42] and Luciano et al. [8] fed lambs with concentrates containing high levels of DCP (24 and 35%) and olive cake (25%), respectively, and observed a reduction in TBARS levels in meat compared with those for lambs fed high-cereal concentrates, indicating an improvement in the oxidative stability of the meat. Inserra et al. [42] attributed this improvement to the greater polyphenol content of the DCP-containing concentrates compared with the control one. In our study, the CON and BYP concentrates contained 1.95 and 6.05 g of gallic equivalents (GAE)/kg DM, respectively, but no differences between groups in the TBARS concentrations were detected when data were analyzed as repeated measures. However, TBARS levels after 6 days of storage were 2.4 times greater for CON than for BYP-fed lambs, and when these data were analyzed independently, differences between groups reached the significance level (*p* = 0.026; SEM = 0.0720; n = 12). In agreement with previous studies [8,42,53], the TBARS concentrations increased (*p* < 0.001) with storage time, but whereas, in the CON lambs, values increased from day 3 to day 6, TBARS concentrations in BYP lambs remained stable from day 3 thereafter. These results would indicate greater oxidative stability of the meat from the BYP-fed lambs after 6 days of storage. It should be noticed that TBARS concentrations in all samples were below 0.5 mg MDA/kg meat, which is the proposed threshold for the detection of off-flavors (rancidity) by a trained sensory panel [56].

As shown in Figure 2, concentrations of CD were not affected either by the diet (*p* = 0.219) or the storage time (*p* = 0.642), and no diet × time interaction was detected (*p* = 0.740). The PUFA are preferential substrates for lipid oxidation in the muscle [57], and in our study, feeding the BYP concentrate resulted in greater deposition of PUFA in the meat (*p* = 0.003; Table 5) compared to CON lambs. An increase in PUFA concentration can negatively influence the oxidative stability of meat, as the FA susceptibility to oxidation increases with increasing unsaturation degree [58]. The lack of differences between diets in CD concentrations in our study might be due to the small differences in total PUFA concentration in the meat after slaughter (9.23 and 10.5% of total FA for CON and BYP meat, respectively). In addition, the concentrations of CD in the meat of both groups remained stable over the storage period, indicating that no extensive PUFA oxidation took place. This is in agreement with the lack of reduction in the proportion of any of the PUFA analyzed (Table 5). In contrast, the proportion of total monounsaturated FA (MUFA) decreased (*p* = 0.035) after 6 days of storage, which is in agreement with the increased TBARS concentrations in the meat of both groups at day 6, as malondialdehyde is an end product of both MUFA and PUFA peroxidation [59]. Our results are in accordance with those of Luciano et al. [8], who analyzed the effects of feeding lambs with a concentrate containing 25% olive cake to lambs and who observed stronger differences between experimental treatments in TBARS than in CD concentrations in the meat.

Ruminant meat is usually regarded as less healthy for humans than the meat of other species such as poultry and pig, which is mainly due to its high content in SFA [9]. The differences in the FA profile of the two concentrates used in our study were reflected in the meat FA profile, as lambs fed the BYP concentrate had lower proportions of total SFA (*p* = 0.001) and greater of total MUFA (*p* = 0.035) and PUFA (*p* = 0.021) than those fed the CON one. There were also differences between groups in the individual FA, and the meat from BYP-fed lambs had lower (*p* ≤ 0.026) proportions of C15:0, C16:0, and C17:0 and tended to greater proportions of C20:0 (*p* = 0.089). Similarly, proportions of C16:1 n-9, C20:1 n-9, C18:2 n-6, C18:3 n-3, and C18:4 n-3 were greater (*p* ≤ 0.036) in the BYP compared with CON lambs. Similar results were obtained by others when lambs were fed concentrates containing either corn DDGS [60] or dried citrus pulp [9,61,62]. The lack of differences (*p* = 0.251) between groups in the proportion C18:1 n-9 observed in our study agrees with the results of Kotsampasi et al. [50], who did not observe differences in this FA in lambs fed destoned EOC, and it was attributed to both the low fat content of the EOC (2.91%, DM basis) and the low level of inclusion in the BYP concentrate (8%). In contrast, several studies have reported increases in the proportion of C18:1 n-9 in the meat of lambs by feeding crude olive cake [63,64]. In agreement with previous results in lambs fed high-concentrate diets [34,48,65], for both groups of lambs, the most abundant FA was (C18:1 n-9), followed by C16:0 and C18:0. Although there were no differences (*p* = 0.157) between groups in the peroxidability index of the meat, the meat from BYP-fed lambs had lower (*p* ≤ 0.003) values in the atherogenic and thrombogenic indexes and a greater (*p* < 0.001) hypocholesterolaemic/hypercholesterolaemic ratio (hH), altogether indicating a healthier FA profile compared with CON lambs. Finally, it should be noticed that the column used for the FA analysis did not allow precise identification of *cis* and *trans* isomers of unsaturated FA [66], and therefore, differences between groups in the meat FA profile are limited to the FA identified.

The storage of meat for 6 days resulted in subtle changes in the SFA and MUFA profiles, as the proportions of C14:0 tended to decrease (*p* = 0.088) and those of C20:1 n-9 increased (*p* = 0.009), with no changes detected for other SFAs and MUFAs. In contrast, the proportions of most of the analyzed PUFAs were affected by meat storage. The proportions of C18:2 n-6, C20:3 n-6, C20:4 n-6, C22:4 n-6, and C22:6 n-3 were increased (*p* ≤ 0.036) and those of C18:3 n-3 and C18:4 n-3 were reduced (*p* = 0.043 and 0.076, respectively) after 6 days of storage, resulting in greater (*p* = 0.021) PUFA proportions in the meat compared with day 0. Whereas no diet × time interaction was observed for any SFA and MUFA, significant interactions (*p* ≤ 0.049) were detected for C20:3 n-6, C20:5 n-3, and C22:4 and trends (*p* ≤ 0.095) were detected for C18:2 n-6, C20:4 n-6, and C22:6 n-3. Majewska et al. [70] also observed similar changes after refrigerated storage of lamb meat for 1 month, which were attributed to the hydrolytic processes and changes in chemical composition occurring during storage. In contrast, a reduction in the proportions of PUFA as storage time increased has been reported by others [71,72], especially when meat contained high proportions of PUFA and was packed in a high-oxygen modified atmosphere. Changes in the FA profiles of meat depend not only on the FA composition and saturation degree but also on the processing methods, storage conditions (temperature, time, atmosphere, etc.), and the concentrations of pro and antioxidants [73]. The peroxidability index increased (*p* = 0.013) over the storage period, but the rest of indexes were not affected by storage.

There were no differences (*p* ≥ 0.616) between groups in the color parameters of the subcutaneous fat of the tail root (Table 6), but the FA profile was markedly affected by diet. The proportion of C16:0 and C17:0 was lower (*p* ≤ 0.011) in the BYP-fed lambs than in those fed the CON diet, resulting in a lower proportion (*p* = 0.001) of total SFA. C16:1 n-9 and C17:1 were the only MUFAs affected by the diet, and their proportion increased for C16:1 n-9 (*p* = 0.008) and tended to decrease for C17:1 (*p* = 0.076) by feeding the BYP concentrate. The greatest difference between diets was observed in the PUFA, as all PUFA proportions were greater (*p* ≤ 0.019) in the BYP-fed lambs compared with those fed the CON concentrate. As a consequence, the proportion of total PUFA in the subcutaneous fat of the tail root was 1.5 times greater (*p* < 0.001) in the BYP group than in the CON one, and the fat from BYP lambs had a greater (*p* < 0.001) peroxidability index. As reported in previous studies in lambs [48,67,74], changes in FA profile induced by diet were greater in the subcutaneous than in the intramuscular fat. As discussed by Manso et al. [48], this can be attributed to the greater deposition rate of the subcutaneous fat compared with the intramuscular fat during the finishing period and to the fact that subcutaneous fat is more responsive to changes in dietary FA supply [75]. Similarly to that observed for the meat, there were no differences (*p* = 0.322) between groups in the peroxidability index of the fat but the fat from BYP-fed lambs had a healthier FA profile as indicated by the lower (*p* ≤ 0.012) atherogenic and thrombogenic indexes and the greater (*p* = 0.002) hH ratio.

## 4. Conclusions

Our results indicate that 44% of cereal grains and protein feeds in the concentrate for fattening light lambs can be replaced with a mixture of corn DDGS, DCP, and EOC without affecting the pH, chemical composition, and color of the meat. However, the use of this mixture changed the FA content of subcutaneous and intramuscular fat to a healthier profile, which has practical implications for human health. In addition, lipid oxidation after 6 days of refrigerated storage was reduced, possibly due to the polyphenols supplied by the by-products, indicating that feeding the BYP concentrate to lambs can help to increase the shelf life of the meat. The use of locally produced by-products in lamb feeding would also contribute to circular economy and to lowering the carbon footprint of meat.

## Figures and Tables

**Table 1:** Ingredients, chemical composition, and fatty acid (FA) profiles of experimental concentrates ^1^.

Item	CON	BYP
Ingredients (% as fed)		
Corn	33.0	26.8
Barley	20.0	-
Wheat	10.0	10.0
Soybean meal 47%	12.2	10.2
Palm meal	8.8	-
Colza meal	2.5	2.5
Wheat bran	10.0	3.0
Dry citrus pulp	-	18.0
Corn DDGS	-	18.0
Olive cake	-	8.0
Others ^2^	3.5	3.5
Chemical composition (%, as fed basis)		
Dry matter	89.7	88.6
Ashes	4.82	5.97
Crude protein	16.2	17.5
Ether extract	3.75	6.44
Neutral detergent fiber	19.2	19.5
Acid detergent fiber	7.47	9.31
Acid detergent lignin	1.79	2.80
Total soluble polyphenols ^3^	0.175	0.536
FA profile (g/100 g total FA) ^4^		
C12:0	7.36	0.296
C14:0	2.86	0.382
C16:0	22.5	21.4
C16:1 n-9	0.172	0.002
C18:0	2.90	2.89
C18:1 n-9	28.4	36.4
C18:2 n-6	35.1	37.8
C20:0	0.350	0.436

^1^ Ingredients and chemical composition were reported by de Evan et al. [4]. ^2^ For both concentrates: 1.2% calcium soap, 1.0% calcium carbonate, 0.8% sodium bicarbonate, 0.3% NaCl, and 0.2% vitamin-mineral premix. ^3^ Expressed as gallic acid equivalents. ^4^ All isomers of each monounsaturated FA are included. CON—high-cereal concentrate; BYP—agro-industrial by-products.

**Table 2:** Values of pH of longissimus dorsi and semitendinosus muscles at 0 and 24 h post-slaughter in fattening light lambs fed either a high-cereal concentrate (CON) or a concentrate including by-products (BYP) ^1^.

		Time			*p* =		
Muscle	Concentrate	0 h	24 h	SEM ^2^	Concentrate	Time	Concentrate × Time
Longissimus dorsi	CON	6.67	5.88	0.010	0.427	<0.001	0.881
	BYP	6.63	5.81				
Semitendinosus	CON	6.28	5.89	0.011	0.183	<0.001	0.597
	BYP	6.18	5.82				

^1^ The BYP concentrate contained 18% corn dried distiller grains with solubles (DDGS), 18% dried citrus pulp, and 8% exhausted olive cake (as-fed basis). ^2^ Standard error of the mean.

**Table 3:** Evolution of color parameters of the longissimus dorsi of light lambs fed either a high-cereal concentrate (CON) or a concentrate including by-products (BYP) during storage at 2 °C ^1^.

		Time (days)					*p* =		
Item	Diet	0	3	6	EEM_D_^2^	EEM_T_^2^	Diet	Time	Diet × Time
Color									
L*	CON	55.4 ^a^	57.0 ^b^	56.3 ^ab^	0.25	0.30	0.895	0.006	0.942
	BYP	55.8 ^a^	57.1 ^b^	56.3 ^ab^					
a*	CON	11.7 ^a^	13.9 ^b^	13.4 ^b^	0.21	0.26	0.998	<0.001	0.965
	BYP	11.7 ^a^	13.9 ^b^	13.2 ^b^					
b*	CON	15.3 ^a^	18.0 ^b^	17.8 ^b^	0.20	0.24	0.864	<0.001	0.987
	BYP	15.4 ^a^	18.0 ^b^	17.9 ^b^					
C*	CON	19.3 ^a^	22.8 ^b^	22.3 ^b^	0.27	0.33	0.933	<0.001	0.986
	BYP	19.4 ^a^	22.8 ^b^	22.3 ^b^					
h*	CON	52.7	52.4	53.3	0.30	0.37	0.928	0.142	0.959
	BYP	52.8	52.4	53.6					
Myoglobin (%)	CON	61.9 ^b^	17.1 ^a^	15.3 ^a^	1.41	1.73	0.130	<0.001	0.667
	BYP	56.3 ^b^	15.4 ^a^	13.4 ^a^					
Metmyoglobin (%)	CON	15.8 ^a^	26.9 ^b^	34.8 ^c^	0.36	0.44	0.592	<0.001	0.881
	BYP	15.8 ^a^	30.2 ^b^	35.3 ^c^					
Oxymyoglobin (%)	CON	22.3 ^a^	53.3 ^b^	48.9 ^b^	1.45	1.78	0.224	<0.001	0.608
	BYP	27.9 ^a^	54.4 ^b^	51.3 ^b^					

^a,b,c^ Within each variable, mean values in the same row with different superscripts differ (*p* < 0.05). ^1^ The BYP concentrate contained 18% corn DDGS, 18% dried citrus pulp, and 8% exhausted olive cake (as-fed basis). ^2^ EEM_D_ and EEM_T:_ standard error of the mean for diet and time effects, respectively.

**Table 4:** Chemical composition and texture parameters of the longissimus dorsi muscle of light lambs fed either a high-cereal concentrate (CON) or a concentrate including by-products (BYP) ^1^.

Item	Concentrate			
	CON	BYP	SEM ^2^	*p =*
Chemical composition (%)				
Moisture	71.7	72.0	1.72	0.636
Protein	22.5	22.6	1.59	0.837
Fat	4.42	4.11	0.228	0.573
Ash	1.28	1.29	0.085	0.813
Texture parameters				
Shear firmness (newton/s cm^2^)	15.4	17.1	0.83	0.166
Shear force (newton/cm^2^)	41.1	46.0	2.72	0.209
Total area (newton s/cm^2^)	184	197	10.5	0.301

^1^ The BYP concentrate contained 18% corn DDGS, 18% dried citrus pulp, and 8% exhausted olive cake (as-fed basis). ^2^ Standard error of the mean.

**Table 5:** Fatty acid (FA) profile, peroxidation index, and indexes related to human health of intramuscular fat of the longissimus dorsi muscle of light lambs fed either a high-cereal concentrate (CON) or a concentrate including by-products (BYP) ^1^.

Item	Diet	Day		*p* *=*			
		0	6	SEM ^2^	Diet	Day	Diet × Day
Fatty acid (% of total fatty acids)							
C14:0	CON	3.97	3.94	0.067	0.212	0.088	0.203
	BYP	3.78	3.58				
C15:0	CON	0.34	0.34	0.007	0.026	0.383	0.646
	BYP	0.27	0.27				
C16:0	CON	23.7	23.7	0.095	<0.001	0.220	0.349
	BYP	21.9	22.1				
C17:0	CON	1.20	1.19	0.020	0.006	0.143	0.565
	BYP	0.99	0.95				
C18:0	CON	14.2	14.4	0.17	0.399	0.179	0.056
	BYP	14.0	13.4				
C20:0	CON	0.12	0.12	0.005	0.089	0.377	0.257
	BYP	0.14	0.13				
Total saturated FA (SFA)	CON	43.6	43.7	0.25	0.001	0.279	0.170
	BYP	41.0	40.4				
C14:1 n-5	CON	0.19	0.20	0.004	0.178	0.658	0.153
	BYP	0.18	0.17				
C16:1 n-7	CON	2.25	2.23	0.020	0.077	0.695	0.490
	BYP	2.06	2.06				
C16:1 n-9	CON	0.27	0.29	0.011	0.036	0.257	0.985
	BYP	0.31	0.32				
C17:1	CON	0.76	0.77	0.006	0.004	0.126	0.470
	BYP	0.57	0.59				
C18:1 n-7	CON	5.74	5.58	0.114	0.625	0.950	0.203
	BYP	5.96	6.10				
C18:1 n-9	CON	37.8	37.7	0.235	0.251	0.122	0.237
	BYP	39.2	38.5				
C20:1 n-9	CON	0.18	0.19	0.004	0.002	0.009	0.542
	BYP	0.21	0.22				
Total monounsaturated FA (MUFA) ^3^	CON	47.2	46.9	0.16	0.085	0.035	0.450
	BYP	48.5	48.0				
C18:2 n-6	CON	6.13	6.20	0.134	<0.001	0.036	0.095
	BYP	7.60	8.13				
C18:3 n-3	CON	0.28	0.27	0.002	0.000	0.043	0.817
	BYP	0.33	0.33				
C18:4 n-3	CON	0.16	0.16	0.002	0.002	0.076	0.339
	BYP	0.22	0.21				
C20:2 n-6	CON	0.28	0.28	0.011	0.586	0.112	0.139
	BYP	0.28	0.31				
C20:3 n-6	CON	0.13	0.14	0.006	0.752	0.017	0.049
	BYP	0.12	0.15				
C20:4 n-6	CON	1.62	1.70	0.092	0.834	0.015	0.090
	BYP	1.42	1.83				
C20:5 n-3	CON	0.10	0.10	0.002	0.614	0.617	0.003
	BYP	0.09	0.10				
C22:4 n-6	CON	0.28	0.30	0.012	0.465	0.001	0.019
	BYP	0.27	0.34				
C22:5 n-3	CON	0.02	0.01	0.001	0.804	0.390	0.202
	BYP	0.01	0.01				
C22:6 n-3	CON	0.22	0.23	0.007	0.258	0.001	0.071
	BYP	0.19	0.23				
Total polyunsaturated FA (PUFA) ^3^	CON	9.23	9.39	0.258	0.003	0.021	0.076
	BYP	10.5	11.6				
Indexes							
Peroxidability index ^4^	CON	19.1	19.6	0.62	0.157	0.013	0.064
	BYP	19.7	22.6				
Atherogenic index ^5^	CON	0.71	0.70	0.009	<0.001	0.276	0.401
	BYP	0.63	0.61				
Thrombogenic index ^5^	CON	1.14	1.34	0.015	0.003	0.157	0.185
	BYP	1.02	0.98				
hH ratio ^6^	CON	1.68	0.15	0.017	<0.001	0.785	0.703
	BYP	1.90	1.92				

^1^ The BYP concentrate contained 18% corn DDGS, 18% dried citrus pulp, and 8% exhausted olive cake (as-fed basis). ^2^ Standard error of the mean. ^3^ All isomers of each unsaturated FA are included. ^4^ Calculated as proposed by Arakawa and Sagai [67]: peroxidability index = (% monoenoic × 0.025) + (% dienoic × 1) + (% trienoic × 2) + (% tetraenoic × 4) + (% pentaenoic × 6) + (% hexaenoic × 8). ^5^ Calculated as proposed by Ulbricht and Southgate [68]: atherogenic index = (C12:0 + 4 × C14:0 + C16:0)/(MUFA + n-6 PUFA + n-3 PUFA) and thrombogenic index = (C14:0 + C16:0 + C18:0)/(0.5 × MUFA + 0.5 × n-6 PUFA + 3 × n-3 PUFA + (n-3/n-6)). ^6^ Hypocholesterolaemic/hypercholesterolaemic ratio calculated as proposed by Santos-Silva et al. [69]: ((C18:1 n-9 + C18:2 n-6 + C20:4 n-6 + C18:3 n-3 + C20:5 n-3 + C22:5 n-3 + C22:6 n-3)/(C14:0 + C16:0)).

**Table 6:** Color parameters, fatty acid (FA) profile, peroxidation index, and indexes related to human health of the subcutaneous fat of the tail root of light lambs fed either a high-cereal concentrate (CON) or a concentrate including by-products (BYP) ^1^.

	Concentrate			
Item	Control	BYP	SEM ^2^	*p* =
Color				
Lightness (L*)	76.7	76.4	0.88	0.764
Redness (a*)	3.53	3.59	0.363	0.911
Yellowness (b*)	14.1	14.6	0.72	0.616
Chromaticity (C*)	14.5	15.1	0.77	0.626
Hue (h*)	76.0	76.6	0.96	0.701
FA (% of total FA)				
C14:0	4.55	4.40	0.192	0.605
C15:0	0.70	0.62	0.060	0.341
C16:0	23.6	22.0	0.33	0.003
C17:0	2.00	1.53	0.120	0.011
C18:0	10.79	9.73	0.564	0.196
C20:0	0.11	0.11	0.005	0.459
Total saturated FA	41.7	38.4	0.62	0.001
C14:1 n-5	0.95	0.82	0.084	0.280
C16:1 n-7	2.73	2.87	0.108	0.379
C16:1 n-9	0.29	0.37	0.019	0.008
C17:1	1.36	1.11	0.096	0.076
C18:1 n-7	6.75	6.59	0.675	0.871
C18:1 n-9	41.4	42.7	0.87	0.294
C20:1 n-9	0.21	0.21	0.007	0.559
Total monounsaturated FA	53.7	54.7	0.65	0.284
C18:2 n-6	3.83	5.88	0.236	<0.001
C18:3 n-3	0.29	0.37	0.015	0.001
C18:4 n-3	0.20	0.31	0.024	0.005
C20:2 n-6	0.12	0.15	0.004	<0.001
C20:3 n-6	0.03	0.04	0.002	0.019
C20:4 n-6	0.12	0.19	0.010	<0.001
Total polyunsaturated FA ^3^	4.60	6.94	0.260	<0.001
Indexes				
Peroxidability index ^4^	7.24	10.20	0.322	<0.001
Atherogenic index ^5^	0.72	0.64	0.019	0.012
Thrombogenic index ^5^	1.05	0.90	0.027	0.001
hH ratio ^6^	1.63	1.87	0.049	0.002

^1^ The BYP concentrate contained 18% corn DDGS, 18% dried citrus pulp, and 8% exhausted olive cake (as-fed basis). ^2^ Standard error of the mean. ^3^ All isomers of each unsaturated FA are included. ^4^ Calculated as proposed by Arakawa and Sagai [67]: peroxidability index = (% monoenoic × 0.025) + (% dienoic × 1) + (% trienoic × 2) + (% tetraenoic × 4) + (%pentaenoic × 6) + (% hexaenoic × 8). ^5^ Calculated as proposed by Ulbricht and Southgate [68]: atherogenic index = (C12:0 + 4 × C14:0 + C16:0)/(MUFA + n-6 PUFA + n-3 PUFA) and thrombogenic index = (C14:0 + C16:0 + C18:0)/(0.5 × MUFA + 0.5 × n-6 PUFA + 3 × n-3 PUFA + (n-3/n-6)). ^6^ Hypocholesterolaemic/hypercholesterolaemic ratio calculated as proposed by Santos-Silva et al. [69]: ((C18:1 n-9 + C18:2 n-6 + C20:4 n-6 + C18:3 n-3 + C20:5 n-3 + C22:5 n-3 + C22:6 n-3)/(C14:0 + C16:0)).
